# Epigenetic Clocks Are Not Accelerated in COVID-19 Patients

**DOI:** 10.3390/ijms22179306

**Published:** 2021-08-27

**Authors:** Julia Franzen, Selina Nüchtern, Vithurithra Tharmapalan, Margherita Vieri, Miloš Nikolić, Yang Han, Paul Balfanz, Nikolaus Marx, Michael Dreher, Tim H. Brümmendorf, Edgar Dahl, Fabian Beier, Wolfgang Wagner

**Affiliations:** 1 Helmholtz-Institute for Biomedical Engineering, Stem Cell Biology and Cellular Engineering, Medical Faculty of RWTH Aachen University, 52074 Aachen, Germany; jufranzen@ukaachen.de (J.F.); snuechtern@ukaachen.de (S.N.); vtharmapalan@ukaachen.de (V.T.); nikolic.m.milos@hotmail.com (M.N.); yang.han@rwth-aachen.de (Y.H.); 2 Department of Hematology, Oncology, Hemostaseology and Stem Cell Transplantation, University Hospital Aachen, RWTH Aachen University, 52074 Aachen, Germany; mvieri@ukaachen.de (M.V.); tbruemmendorf@ukaachen.de (T.H.B.); fbeier@ukaachen.de (F.B.); 3 Center for Integrated Oncology Aachen Bonn Cologne Düsseldorf (CIO ABCD), 52074 Aachen, Germany; 4 Department of Cardiology, Angiology and Intensive Care Medicine (Department of Internal Medicine I), University Hospital Aachen, RWTH Aachen University, 52074 Aachen, Germany; pbalfanz@ukaachen.de (P.B.); nmarx@ukaachen.de (N.M.); 5 Department of Pneumology and Intensive Care Medicine, University Hospital Aachen, RWTH Aachen University, 52074 Aachen, Germany; mdreher@ukaachen.de; 6 Institute of Pathology, University Hospital Aachen, RWTH Aachen University, 52074 Aachen, Germany; edahl@ukaachen.de; 7 RWTH Centralized Biomaterial Bank (RWTH cBMB), Medical Faculty of RWTH Aachen University, 52074 Aachen, Germany

**Keywords:** age, COVID-19, DNA methylation, epigenetic clocks, SARS-CoV-2, telomere

## Abstract

Age is a major risk factor for severe outcome of the 2019 coronavirus disease (COVID-19). In this study, we followed the hypothesis that particularly patients with accelerated epigenetic age are affected by severe outcomes of COVID-19. We investigated various DNA methylation datasets of blood samples with epigenetic aging signatures and performed targeted bisulfite amplicon sequencing. Overall, epigenetic clocks closely correlated with the chronological age of patients, either with or without acute respiratory distress syndrome. Furthermore, lymphocytes did not reveal significantly accelerated telomere attrition. Thus, these biomarkers cannot reliably predict higher risk for severe COVID-19 infection in elderly patients.

## 1. Introduction

The clinical manifestation of COVID-19 is very heterogeneous. Some patients remain asymptomatic or have only mild symptoms throughout the course of infection, whereas others experience severe disease with hospitalization or even death [1]. There is a need to better identify persons that are at higher risk for poor outcome of the COVID-19 to facilitate even better protection and early treatment. Chronological age is one of the major risk factors for developing severe symptoms during an infection with severe acute respiratory syndrome coronavirus 2 (SARS-CoV-2) [2], and this risk is independent of other age-related comorbidities [3]. The estimated infection fatality rate (IFR) is very low in children and younger adults but increases exponentially with age [2]. It is conceivable that biological age provides an even better measure for risk assessment than chronological (or calendarian) age [4]. Unfortunately, despite intensive research, there is no commonly accepted specific measure for biological age [5]. The process of biological aging is reflected by molecular hallmarks, which include epigenetic modifications. Age-associated epigenetic modifications are particularly reflected by highly reproducible DNA methylation changes at specific CG dinucleotides (CpG sites). The DNA methylation levels of several age-associated CpGs can be combined into “epigenetic clocks” to predict donor age [6]. There is evidence that the epigenetic age of blood does not only reflect chronological age but also aspects of biological age; an increased epigenetic age is associated with higher all-cause mortality and higher risk for various age-associated comorbidities [6,7]. We therefore followed the hypothesis that accelerated epigenetic age also increases susceptibility to severe COVID-19 infections that require hospitalization [3,8].

## 2. Results

In this study, we used blood samples of 50 hospitalized patients with severe SARS-CoV-2 infection, with or without acute respiratory distress syndrome (ARDS) (Supplementary Table S1). In addition, we used available DNA methylation profiles generated with the Illumina EPIC BeadChips of 102 hospitalized COVID-19 patients (GSE174818) [9], of 407 COVID-19 patients with both mild or severe symptoms (GSE168739) [10], and of 9 COVID-19 patients from our hospital (GSE161988). For comparison, we utilized datasets of healthy donors, which were generated before the pandemic (GSE123914, GSE42861, and GSE61496). Principle component analysis (PCA) of the first two principal components described nearly 100% of variability between the datasets and revealed only a moderate difference between the datasets after background correction (Supplementary Figure S1). For orientation, we estimated the cellular composition of leucocytes based on DNA methylation profiles [11]. This analysis showed reduced fractions of lymphocyte subsets (CD4, CD8, NK and B-cells) and increased monocytes and granulocytes in COVID-19 patients, which is in line with previous and own measurements with flow cytometry (Supplementary Figure S2) [12]. 

**Figure 1:**
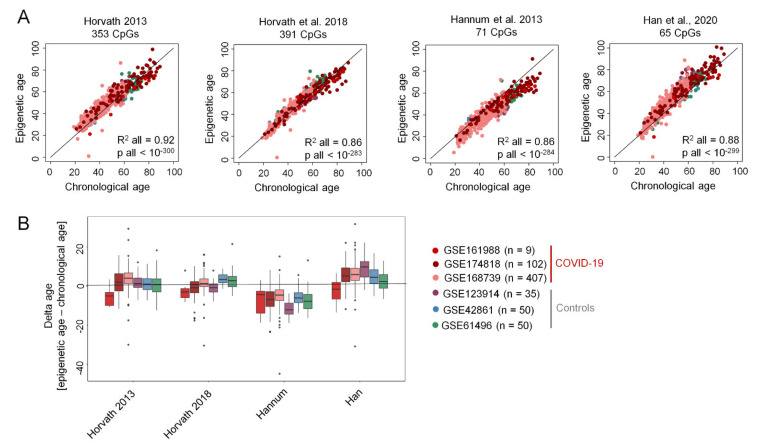
Epigenetic ageing clocks based on Illumina BeadChip data are not accelerated in COVID-19 patients. (**A**) Genome wide DNA methylation profiles of COVID-19 patients from three different studies (GSE161988; GSE174818; and GSE168739), as well as 135 controls of healthy donors of three studies (GSE123914, GSE42861; GSE61496) were analyzed with four different predictors of epigenetic age as described by Horvath 2013 [13], Horvath et al. 2018 [14], Hannum et al. 2013 [15], and Han et al. 2020 [16]. For all datasets combined, the coefficient of determination (R^2^) and the regression *p* value are indicated for each predictor. (**B**) Boxplots present the deviation of epigenetic age prediction and chronological age (delta-age). Significance of pairwise comparisons is provided in the Supplementary Figure S3.

We estimated epigenetic age for the Illumina BeadChip profiles with four different predictors: (1) an aging signature for multiple tissues by Horvath [13]; (2) a skin and blood clock of Horvath et al. [14]; (3) an aging signature for blood by Hannum et al. [15]; and (4) our recently described age-predictor for blood [16]. With all four signatures the epigenetic age-predictions of COVID-19 samples correlated clearly with chronological age (Figure 1A). Notably, COVID-19 samples did not consistently reveal accelerated epigenetic age as compared to chronological age (delta-age), while there was some variation between the different predictors and studies (Figure 1B; Supplementary Figure S3). This exemplifies that a targeted assay for epigenetic age-predictions might be advantageous for a direct comparison of delta-age in hospitalized COVID-19 patients and healthy controls.

**Figure 2:**
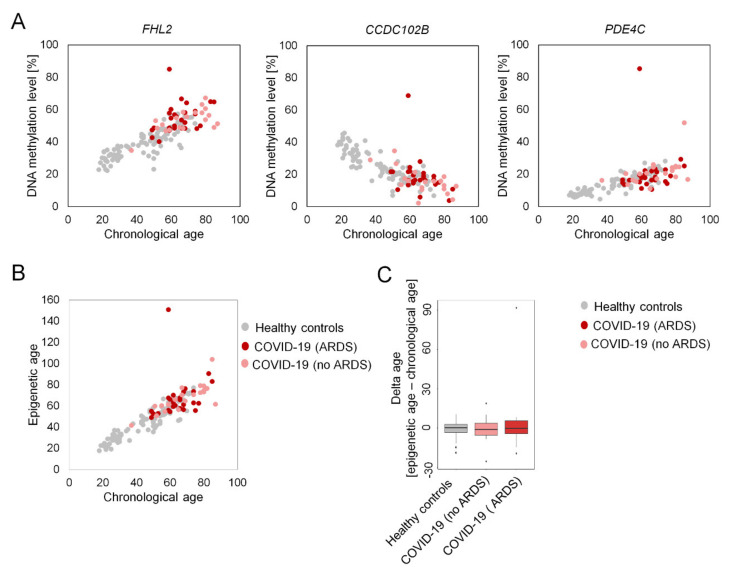
Epigenetic age predictions with amplicon sequencing. (**A**) DNA methylation levels at the 3 age-associated CpG sites correlate with donor age. Samples of 47 COVID-19 patients with (*n* = 27, red) or without ARDS (*n* = 20, light red) do not show an offset in the DNA methylation levels in comparison to 95 healthy control samples (grey), except for one outlier. (**B**) Based on these DNA methylation levels the epigenetic age was predicted with a multivariable model. (**C**) The deviation of chronological and predicted epigenetic age (delta-age) is presented (no significant differences; Welch’s *t* test).

We therefore additionally tested for age-acceleration with targeted bisulfite amplicon sequencing (BA-seq) of three age-associated regions. The relevant CpG sites are associated with the genes Coiled-Coil Domain-Containing Protein 102B (*CCDC102B*), Four And A Half LIM Domains Protein 2 (*FHL2*) and Phosphodiesterase 4C (*PDE4C*), as described before [16]. We validated this method with 95 blood samples of healthy donors (18–74 years) that were collected before the begin of the SARS-CoV-2 pandemic (mean absolute error = 4.19 years; R^2^ = 0.88). There was no evidence for accelerated epigenetic aging in 47 analyzed COVID-19 samples, even if we stratified into samples with or without ARDS (Figure 2). Only one outlier was significantly overestimated (151 years).

**Figure 3:**
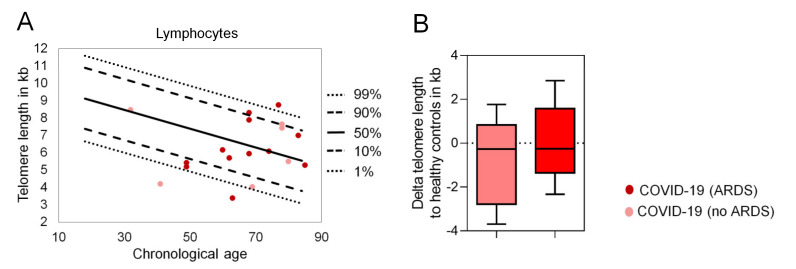
Telomere length analysis in COVID-19 patients. (**A**) Telomere lengths in kilo bases of 19 COVID-19 patients. Bovine thymocytes were used as internal controls. All measurements were carried out in triplicates. Lines indicate percentiles of the telomere lengths of 356 healthy controls. (**B**) Telomere length difference of no ARDS and ARDS patients to the respective age-adapted telomere length.

Telomere attrition is another hallmark of aging and correlates with age in peripheral blood cells. A recent study indicated that in leukocytes of COVID-19 patients telomere attrition below the 10th percentile is more frequent than in healthy controls [17], however the results of this study might be affected by the lymphopenia observed in COVID-19 patients. We analyzed telomere length in lymphocytes of 19 COVID-19 patients with Flow-FISH and, in comparison to 356 healthy controls, there was overall no evidence for significant telomere attrition in COVID-19 patients (Figure 3).

## 3. Discussion

Taken together, our results do not provide evidence that severe outcome of COVID-19 is associated with accelerated epigenetic age or significantly shortened telomere length. This is in contrast to a recent study that indicated that epigenetic age was accelerated in patients with severe COVID-19 infections [8]. The discrepancy might be attributed to the even smaller sample number of COVID-19 samples in the other study (*n* = 9) and to the application of epigenetic clocks that were specifically trained to capture changes in the leukocyte composition [8], which is clearly affected by the disease. On the other hand, there have been epigenome-wide association studies with COVID-19 severity that clearly demonstrate specific DNA methylation changes that are directly linked to the disease [9,10]. Despite such changes in the DNA methylation landscape of COVID-19 patients, our analysis with four aging-signatures and different available datasets demonstrate that epigenetic clocks are not generally affected in patients with severe outcome.

Our study has several limitations: (1) it is conceivable that the SARS-CoV 2 infection itself impacts on the epigenetic age. In fact, it has been demonstrated that HIV infection leads to an average aging advancement of 4.9 years [18]. It has also been suggested that such off-set occurs also if the infection with SARS-CoV-2 has been persistent for a longer time in COVID-19 survivors, particularly in a cohort younger than 60 years [19]. However, blood samples that were taken before a fatal COVID-19 infection were not available, and it appears unlikely that the COVID-19 infection rejuvenates a previously accelerated epigenetic age; (2) aging is a very heterogeneous process—inter-individually, but also intra-individually. It is still unclear if the different tissues of a human organism reveal the same pace of epigenetic aging. Hence, it is conceivable that nasopharyngeal and bronchial epithelium, which is preferentially infected by SARS-CoV-2, reveals higher delta-age with epigenetic age-predictions; (3) the number of samples used in our study for the BA-seq analysis and telomere length measurements is still relatively small. However, the findings are consistent with epigenetic clocks that we applied to the larger EPIC BeadChip datasets. (4) Last but not least, it is possible that additional unknown confounding factors exist, such as specific therapies, which may have biased the results. It has been demonstrated that the severity of the disease may be efficiently controlled thanks to developing structured care [20]. To our knowledge, none of these medications have so far been shown to directly affect epigenetic age and we therefore do not anticipate that the treatments mask a previously existing accelerated epigenetic age. Taken together, our results provide evidence that analysis of epigenetic age in blood is not suitable to reliably stratify elderly patients that are even more susceptible to severe COVID-19 infections.

## 4. Material and Methods

### 4.1. Blood Samples Used in This Study

Blood samples of COVID-19 patients were taken within the first two weeks after the detection of the SARS-CoV-2 infection of patients at University Hospital of RWTH Aachen. All patient samples were taken after written and informed consent according to the guidelines and specific approval of the study by the local ethics committee (Ethic approval number EK 080/20 for the Covid-19 Aachen study named COVAS; Ethics committee of RWTH Aachen University, University Hospital Aachen, Pauwelsstrasse 30, 52074 Aachen, Germany) and collected into RWTH cBMB, the central biobank of the medical faculty of RWTH Aachen University (Ethic approval number EK 206/09). Blood samples of healthy donors were taken after written and informed consent according to the guidelines and approval of the study by the local ethics committee (EK 041/15; Ethics committee of RWTH Aachen University, University Hospital Aachen, Pauwelsstrasse 30, 52074 Aachen, Germany). These control samples were taken in the years 2018 and 2019, before the initial SARS-CoV-2 outbreak. All venous blood samples were anticoagulated with EDTA and cryopreserved at -80 °C until further analysis.

### 4.2. Analysis of DNA Methylation Microarray Data

Genomic DNA of COVID-19 blood samples was isolated using the Maxwell 16 LEV DNA Blood Kit (Promega, AS1290) in a Maxwell 16 instrument. 1200 ng DNA were bisulfite converted and analyzed with Illumina EPIC BeadChip microarrays at Life&Brain (Bonn, Germany). For comparison we exemplarily selected DNA methylation profiles of 135 peripheral blood samples (50 samples of GSE42861, 50 samples of GSE61496, and 35 samples of GSE123914) from Gene Expression Omnibus (https://www.ncbi.nlm.nih.gov/geo (accessed on 26 August 2021)). Furthermore, we used publicly available data from 102 hospitalized COVID-19 patients (GSE174818) and 407 COVID-19 patients with both mild (*n* = 194) or severe symptoms (*n* = 213, GSE168739).

The Illumina EPIC methylation microarray investigates approximately 850,000 CpG sites across the genome. The idat files of the Illumina BeadChip datasets were background corrected using the single-sample Noob (ssNoob) method provided by R package minfi [21]. Principal component analysis was performed with the R package stats. Age predictions with the signatures of Horvath 2013 [13] and Hannum et al. [15] were performed with the R package wateRmelon [22]. The more recent skin and blood clock of Horvath et al. 2018 was estimated as described in the original publication [14]. Age predictions of Han were performed with DNA methylation levels of 65 CpGs, as described before [16]. The predictors of Horvath (2013) and Hannum et al. (2013) comprise CpGs that were not measured by the EPIC BeadChip (19 and 6, respectively) and this might result in a moderate offset of age-predictions. The results of Illumina BeadChip profiles usually provide relatively robust results for epigenetic signatures [13,16,23]. R^2^ and *p* values of the regressions were estimated with the lm function from R package stats. Predictions of leukocyte subsets were produced with the estimateCellCounts function of the R package minfi [11,24].

### 4.3. Bisulfite Amplicon Sequencing

We have recently described BA-seq for nine CpGs that provide robust and reliable age predictions [16]. To further ease applicability of the method, we have meanwhile refined the signature to focus on the three regions with highest correlation with chronological age and with a combination of hyper- and hypomethylated CpGs to reduce the PCR bias. DNA methylation levels at these three age-associated CpG sites (*FHL2, CCDC102B, PDE4C*) were analyzed by bisulfite amplicon sequencing as described in detail before [16,25]. In brief, genomic DNA of 47 COVID-19 and 95 healthy control samples was bisulfite converted using the EZ DNA methylation kit (Zymo Research). The three relevant genomic regions were amplified by PCR using the PyroMark PCR kit (Qiagen) and primers as described before [16]. Illumina adapters were added by a second PCR and samples were sequenced on an Illumina MiSeq sequencer in 250 bp paired end mode using the V2 nano kit (Illumina). Mean read coverage was 3370 reads for all amplicons and all samples. Alignment of reads to the hg19 genome build and calculation of methylation levels were performed with bismark [26]. We utilized BA-seq data of 40 healthy blood samples of our previous work [16] to derive the following multivariable model:

Epigenetic age (years) = −0.34 DNAm^CCDC102B^ + 0.83 DNAm^FHL2^ + 1.18 DNAm^PDE4C^ + 3.86

### 4.4. Fluorescence In Situ Hybridization (Flow-FISH)

Flow-FISH analysis of telomere length was carried out as previously described [27,28]. Briefly, vital frozen mononuclear cells from peripheral blood were mixed with a FITC labeled telomere specific (CCCTAA)3-peptide nucleic acid FISH probe (Eurogentec, Seraing, Belgium) for DNA-hybridization followed by DNA counterstaining with LDS 751 (Sigma, St. Louis, MO, USA). Bovine thymocytes were used as internal controls. All measurements were carried out in triplicates. For comparison, we used telomere length distribution of 356 healthy controls [29]. Telomere length is given in kilobases (Kb).

## Data Availability

The Illumina EPIC microarray dataset of nine COVID-19 patients that was generated in the present study is accessible at NCBI NIH Gene Expression Omnibus (GEO) under the accession number GSE161988.

## Supplementary Materials

The following are available online at https://www.mdpi.com/article/10.3390/ijms22179306/s1, Combined PDF with Supplementary Figures S1–S3, and Supplementary Table S1.
