# Supplementary material for: Epigenetic Clocks Are Not Accelerated in COVID-19 Patients
Source: Int J Mol Sci. 2021 Aug 27;22(17):9306. doi: 10.3390/ijms22179306 (PMC8431654; doi:10.3390/ijms22179306)
Supplement: Supplementary file 1 [file ijms-22-09306-s001.zip › ijms-1326099-supplementary.pdf]

Supplemental Material

Epigenetic clocks are not accelerated in COVID-19 patients

Julia Franzen, Selina Nüchtern, Vithurithra Tharmapalan, Margherita Vieri, Miloš Nikolić, Yang Han, Paul Balfanz, Nikolaus Marx, Michael Dreher, Tim H. Brümmendorf, Edgar Dahl, Fabian Beier, Wolfgang Wagner

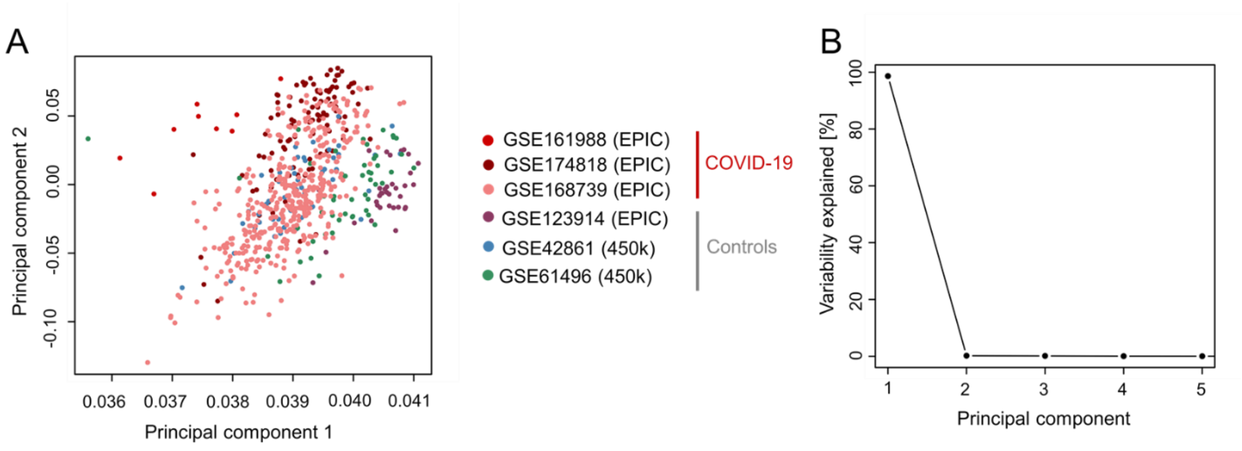

Supplemental Figure S1. Principal component analysis of DNA methylation microarray datasets.

For better comparison we selected up to 50 DNAm profiles per study for the control groups. The corresponding accession numbers for the Gene Expression Omnibus are provided (https://www.ncbi.nlm.nih.gov/geo/). Data were background corrected using the single-sample Noob (ssNoob) method implemented in the R package minfi. Notably, data were generated on either Illumina EPIC or 450k BeadChips, as indicated. We only considered CpG sites that are represented on both platforms and excluded CpGs on Y and X chromosome. **A)** To estimate if there are batch effects between studies, we performed a PCA analysis of DNA methylation datatsets used in this work. The samples cluster weakly according to the different studies, indicating that there are moderate batch effects after background normalization. **B)** PC1 and PC2 describe nearly 100% of the variability between the datasets.

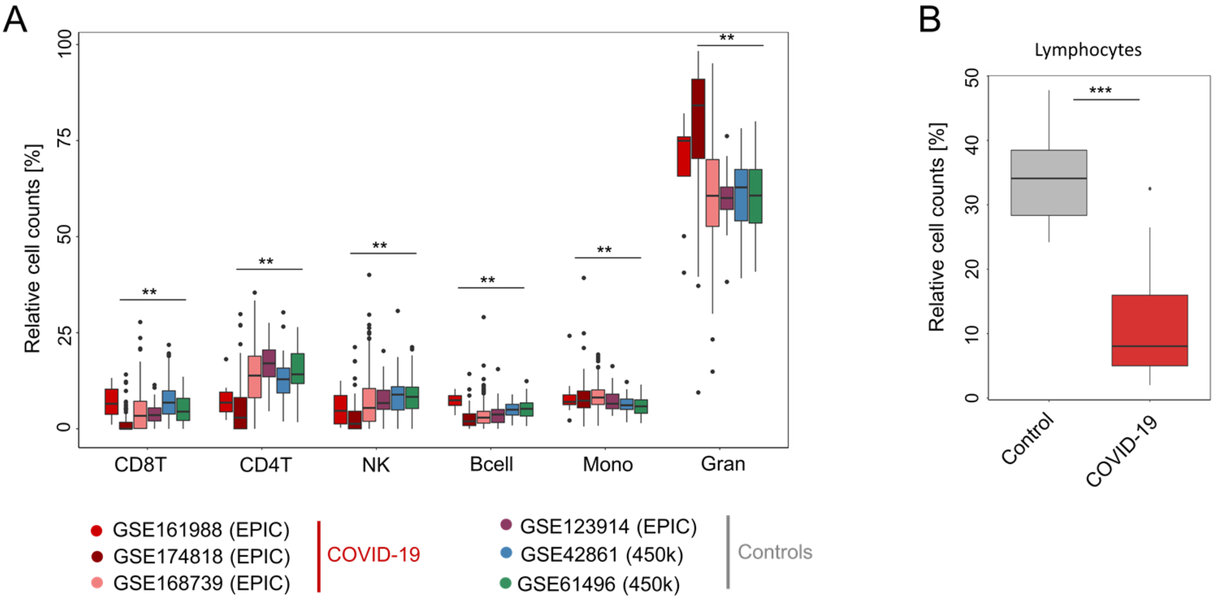

Supplemental Figure S2. Leukocyte counts.

A) The cellular composition of leukocyte subsets was estimated for all DNA methylation profiles using the predictor of Houseman et al. (BMC bioinformatics 2012, 13, 86-86). Welch's t test demonstrated a significant difference for all cell subsets of COVID-19 patients in comparison to healthy controls. (** p-value < 10^{-4}). **B)** Automated cell counting shows a significant decrease of lymphocytes in 18 COVID-19 samples in comparison to 16 healthy controls (** p < 10^{-8}, Welch's t test).

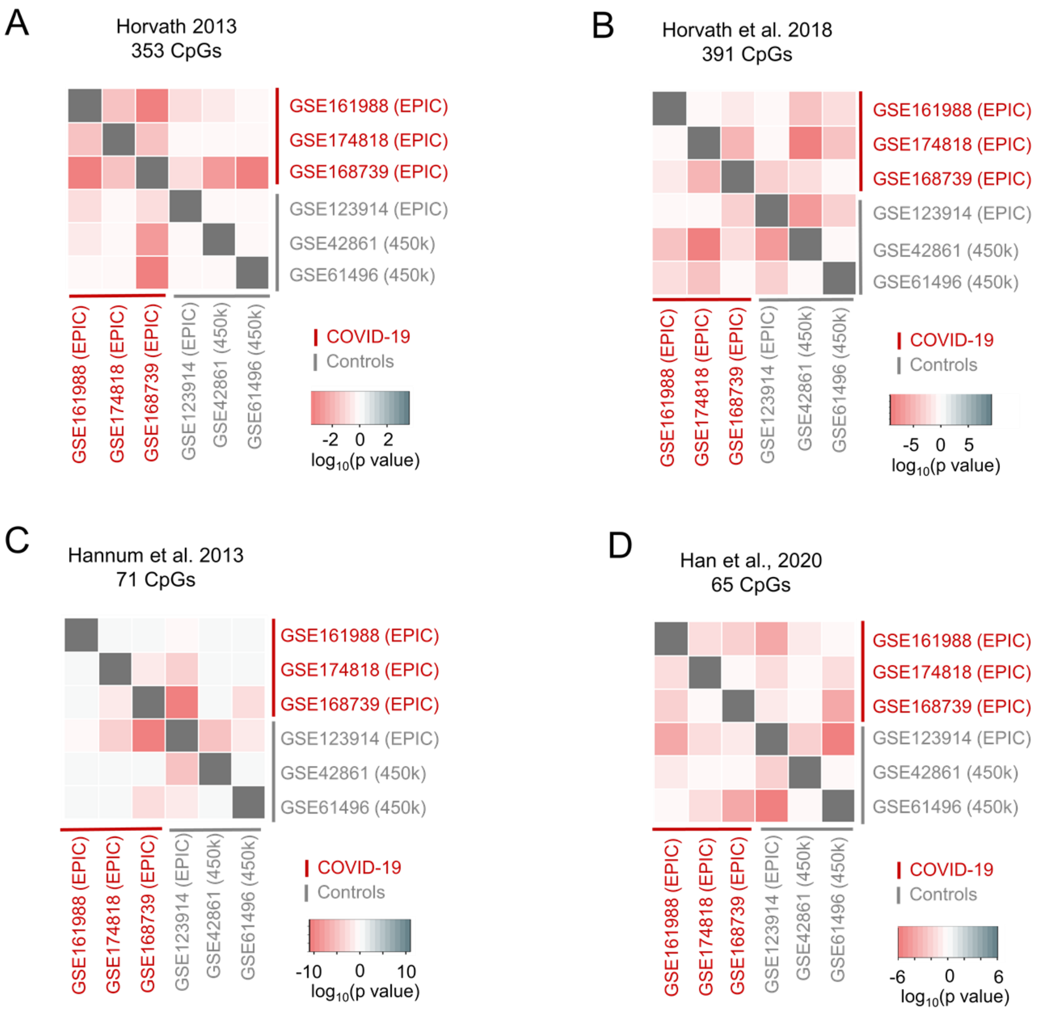

Supplemental Figure S3. Pairwise comparison of delta-age in different studies.

Dunn's test for significance of deviation of predicted and chronological age (delta-age) between all investigated studies as depicted in Figure 1B. The heatmaps show the logarithmic p values of all comparisons for the four different age predictors by **A)** Horvath 2013, **B)** Horvath et al. 2018, **C)** Hannum et al. 2013, and **D)** Han et al. 2020. The figure demonstrates that pairwise comparison of individual studies sometimes provide significant results, but this was not consistently observed for the four different epigenetic aging signatures and sometimes COVID-19 samples were significantly under- or overestimated with different signatures. This exemplifies difficulties of combining DNA methylation profiles of different studies and potential batch effects. In this regard, targeted assays may be more robust.

Supplemental Table S1. Patient characteristics.

Patient ID	Gender	Age range*	Group	EPIC	MiSeq	Flow-FISH
Patient 1	M	80 - 90	no ARDS	x	x	x
Patient 2	M	60 - 70	ARDS	x	x	
Patient 3	F	80 - 90	ARDS	x	x	x
Patient 4	F	70 - 80	ARDS	x	x	x
Patient 5	M	40 - 50	ARDS	x	x	x
Patient 6	M	40 - 50	ARDS	x	x	x
Patient 7	M	60 - 70	ARDS	x	x	x
Patient 8	M	60 - 70	ARDS	x	x	x
Patient 9	F	30 - 40	no ARDS	x		x
Patient 10	M	60 - 70	ARDS		x	x
Patient 11	M	60 - 70	ARDS			x
Patient 12	F	70 - 80	ARDS		x	x
Patient 13	M	60 - 70	no ARDS		x	x
Patient 14	M	80 - 90	ARDS		x	x
Patient 15	F	60 - 70	ARDS		x	x
Patient 16	M	70 - 80	no ARDS		x	x
Patient 17	F	60 - 70	ARDS		x	x
Patient 18	F	60 - 70	no ARDS		x	x
Patient 19	M	70 - 80	no ARDS		x	x
Patient 20	M	40 - 50	no ARDS			x
Patient 21	F	60 - 70	ARDS		x	
Patient 22	F	60 - 70	ARDS		x	
Patient 23	F	60 - 70	ARDS		x	
Patient 24	F	50 - 60	ARDS		x	
Patient 25	M	60 - 70	ARDS		x	
Patient 26	F	70 - 80	ARDS		x	
Patient 27	F	50 - 60	ARDS		x	
Patient 28	M	70 - 80	ARDS		x	
Patient 29	M	50 - 60	ARDS		x	
Patient 30	M	60 - 70	ARDS		x	
Patient 31	M	50 - 60	ARDS		x	
Patient 32	M	50 - 60	ARDS		x	
Patient 33	M	60 - 70	ARDS		x	
Patient 34	M	50 - 60	ARDS		x	
Patient 35	F	60 - 70	ARDS		x	
Patient 36	F	50 - 60	no ARDS		x	
Patient 37	F	50 - 60	no ARDS		x	
Patient 38	M	60 - 70	no ARDS		x	
Patient 39	F	80 - 90	no ARDS		x	
Patient 40	M	60 - 70	no ARDS		x	
Patient 41	F	50 - 60	no ARDS		x	
Patient 42	M	80 - 90	no ARDS		x	
Patient 43	M	80 - 90	no ARDS		x	
Patient 44	F	60 - 70	no ARDS		x	
Patient 45	F	80 - 90	no ARDS		x	
Patient 46	F	50 - 60	no ARDS		x	
Patient 47	M	30 - 40	no ARDS		x	
Patient 48	M	80 - 90	no ARDS		x	
Patient 49	M	50 - 60	no ARDS		x	
Patient 50	F	70 - 80	no ARDS		x	

* Precise age is not provided to prevent patient identifying information.
